# Combining ketamine with astrocytic inhibitor as a potential analgesic strategy for neuropathic pain. ketamine, astrocytic inhibitor and pain

**DOI:** 10.1186/1744-8069-6-50

**Published:** 2010-09-06

**Authors:** Xiao-Peng Mei, Wei Wang, Wen Wang, Chao Zhu, Lei Chen, Ting Zhang, Li-Xian Xu, Sheng-Xi Wu, Yun-Qing Li

**Affiliations:** 1 Department of Anesthesiology, School of Stomatology, Fourth Military Medical University, Xi'an, 710032, China; 2 Department of Anatomy, histology and embryology, K. K. Leung Brain Research Centre, Fourth Military Medical University, Xi'an, 710032, China

## Abstract

**Background:**

Neuropathic pain is an intractable clinical problem. Intrathecal ketamine, a noncompetitive N--methyl-D-aspartate receptor (NMDAR) antagonist, is reported to be useful for treating neuropathic pain in clinic by inhibiting the activity of spinal neurons. Nevertheless, emerging studies have disclosed that spinal astrocytes played a critical role in the initiation and maintenance of neuropathic pain. However, the present clinical therapeutics is still just concerning about neuronal participation. Therefore, the present study is to validate the coadministration effects of a neuronal noncompetitive N-methyl-D-aspartate receptor (NMDAR) antagonist ketamine and astrocytic cytotoxin L-α-aminoadipate (LAA) on spinal nerve ligation (SNL)-induced neuropathic pain.

**Results:**

Intrathecal ketamine (10, 100, 1000 μg/kg) or LAA (10, 50, 100 nmol) alleviated SNL-induced mechanical allodynia in a dose-dependent manner respectively. Phosphorylated NR1 (pNR1) or glial fibrillary acidic protein (GFAP) expression was down-regulated by intrathecal ketamine (100, 1000 μg/kg) or LAA (50, 100 nmol) respectively. The combination of ketamine (100 μg/kg) with LAA (50 nmol) showed superadditive effects on neuropathic pain compared with that of intrathecal administration of either ketamine or LAA alone. Combined administration obviously relieved mechanical allodynia in a quick and stable manner. Moreover, down-regulation of pNR1 and GFAP expression were also enhanced by drugs coadministration.

**Conclusions:**

These results suggest that combining NMDAR antagonist ketamine with an astrocytic inhibitor or cytotoxin, which is suitable for clinical use once synthesized, might be a potential strategy for clinical management of neuropathic pain.

## Background

Neuropathic pain is a formidable clinical problem. Multiple mechanisms are implicated under neuropathic pain, such as superexcited primary afferent, abnormal plasticity in spinal dorsal horn, and aberrant neuron-glia interactions [1-3].

Abundant laboratory and clinical evidence indicates that ketamine, a noncompetitive N--methyl-D-aspartate receptor (NMDAR) antagonist, is useful for treating neuropathic pain [4,5]. The analgesic effect of ketamine is trustful, but undesirable side effects exist, such as dizziness, drowsiness, dry mouth, sedation, loss of appetite, nausea, vomiting and so on [6,7]. The clinical concept of balanced or associative manner proposes to use a combination of analgesics or other treatments to provide better pain relief with minimal side effects [8]. For instance, studies have indicated that ketamine works better when combined with morphine, amitriptyline, or electroacupuncture, respectively [8-10]. All these combinations have well minimized the side effects of ketamine, but still fail to provide an ideal analgesic effect on neuropathic pain. Notably, all of these combinations are just concern about the neuronal participation in the neuropathic pain states.

Accumulating evidence suggests that communication between neurons and glia is essential for neuropathic pain processing [1,11,12]. Studies even indicate that glial activation is required and sufficient for chronic pain sensitization [13-17]. Therefore, spinal glia should be considered when treating neuropathic pain. By releasing neurotransmitters or other extracellular signaling molecules, and reuptaking neurotransmitters among synaptic cleft, glia can affect neuronal excitability, synaptic transmission and perhaps coordinate activity in neuronal networks [1]. The results of previous studies have implicated spinal cord glial cells (especially astrocytes and microglia) as key players in the induction and maintenance of neuropathic pain [18-20]. Astrocytes are one of important cells type for maintenance of spinal nerve ligation (SNL)-induced neuropathic pain [21-23]. Therefore, inhibition of astrocytic activation could help relieve neuropathic pain, which breaks the "cross-talk" between neurons and astrocytes [24].

Taken together, we hypothesized that combination of NMDAR antagonist ketamine and an astrocytic inhibitor might exhibit some additive and complementary effects on neuropathic pain and propose a potential strategy for therapy.

To test this hypothesis, we employed SNL-induced neuropathic pain model. ketamine or astrocytes specific cytotoxin L-α-aminoadipate (LAA) was intrathecally injected to confirm their individual dose-dependent analgesia effect. Based on the dose-effect curve, these two agents were intrathecally coadministrated at the safe dosages. The analgesic effects of the above mentioned treatments were evaluated, while the potential side effects of ketamine on the motor function were observed with rotarod test. Western blot and immunohistochemistry were performed to confirm the effects of these two agents, administered individually or together, on SNL-induced NMDA receptor phosphorylation and astrocytic activation.

## Results

### SNL induced significant mechanical allodynia, astrocytic activation and NMDA receptor phosphorylation

SNL produced rapidly appearing and persistent mechanical allodynia. The paw withdrawal threshold (PWT, absolute threshold) was much lower in the SNL-Saline group than that of Sham-Saline or Naïve group on POD 7. The PWTs of Sham-Saline rats were not different with that of Naïve rats.

**Figure 1:**
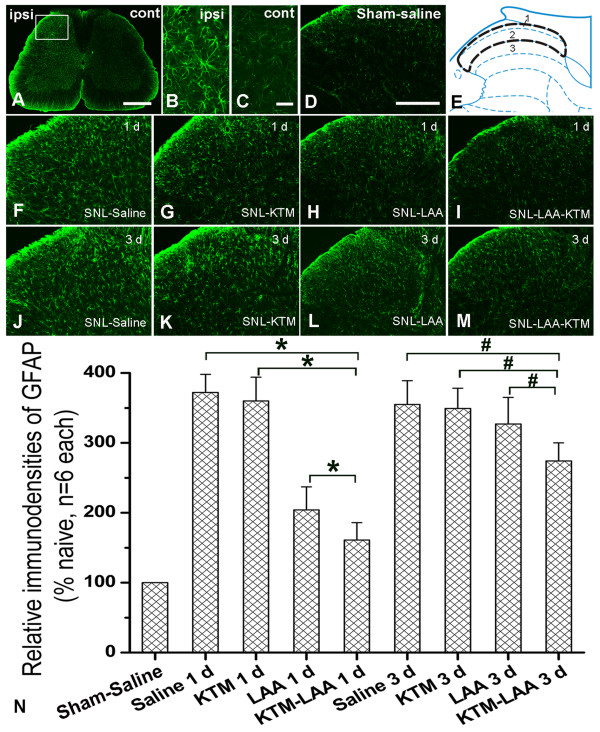
**Effects of drugs administration on spinal nerve ligation (SNL) -induced astrocytic activation**. A representative spinal section showed that SNL induced a marked astrocytic activation in ipsilateral than that of contralateral spinal dorsal horn (A). B and C show the magnified images of the ipsilateral and contralateral spinal dorsal horn of SNL rats respectively. Compared with Sham-Saline rats (D), SNL induced a marked astrocytic activation (F, J). Intrathecal ketamine (100 μg/kg) didn't affect influence astrocytic activation obviously at both 1 d and 3 d after application (G, K). However, intrathecal LAA (50 nmol) attenuated SNL-induced astrocytic activation at 1 d after administration compared with that of SNL-Saline group (H). Astrocytic activation was further suppressed by coadministration (100 μg/kg of ketamine and 50 nmol of LAA) than that of LAA (50 nmol) injected alone (I). Astrocytic activation was returned obvious 3 d after LAA injection (L). However, drugs coadministration significantly inhibited astrocytic activation compared to that of LAA alone (M). E, Scheme presenting an overview over the detected region. N, Statistics of above results. Scalebar = 800 μm in A, 20 μm in B-C and 200 μm in D, F-M. * or # indicates statistically significant difference with *P *< 0.05 between groups. KTM: ketamine, LAA: L-α-aminoadipate.

Besides, SNL induced a marked astrocytic activation, indicated by glial fibrillary acidic protein (GFAP) up-regulation in the ipsilateral spinal dorsal horn of SNL-Saline group (Fig 1A). Immunohistochemistry of GFAP indicated that activated astrocytes showed hypertrophied cell bodies with thickened processes and GFAP-immunoreactive (IR) staining was enhanced (Fig 1B). We have observed in our previous study that the SNL-induced astrocytic activation was not evident on POD 1, but significant on POD 3, reaching a peak on POD 7, and remained at high levels at 3 w after SNL [25].

**Figure 2:**
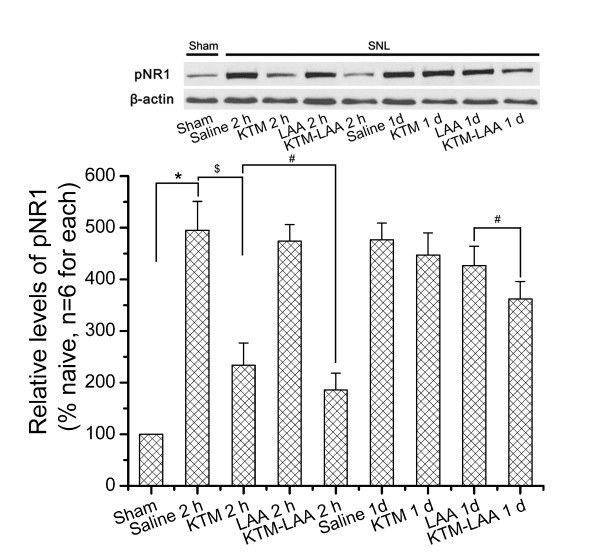
**Effects of drugs administration on SNL-induced NR1 phosphorylation**. Phosphorylated NR1 (pNR1) level was much higher in SNL-Saline group than that of Sham-Saline group (* *P *< 0.05). Intrathecal ketamine (100 μg/kg) down-regulated pNR1 level compared with that of SNL-Saline group. Coadministration further inhibited pNR1 expression than that of individual ketamine injection. Furthermore, pNR1 returned to high level at 1 d after individual ketamine injection. However, pNR1 remained low at 1 d in the coadministration group compared with that of individual ketamine injection. Additionally, LAA application alone has no obvious effects on SNL-induced NR1 phosphorylation. *, # and $ each indicates statistically significant difference with *P *< 0.05 between groups.

NR1 is the essential subunit of NMDAR [26]. The functional change of NMDAR induced by SNL was evaluated by phosphorylation of NR1 in our experiment. Compared to that of Sham-Saline group, the level of anti-phosphorylated NR1 (pNR1) was significantly increased in SNL-Saline group on POD 7 (*P *< 0.05 compared to Sham-Saline) (Fig 2).

**Figure 3:**
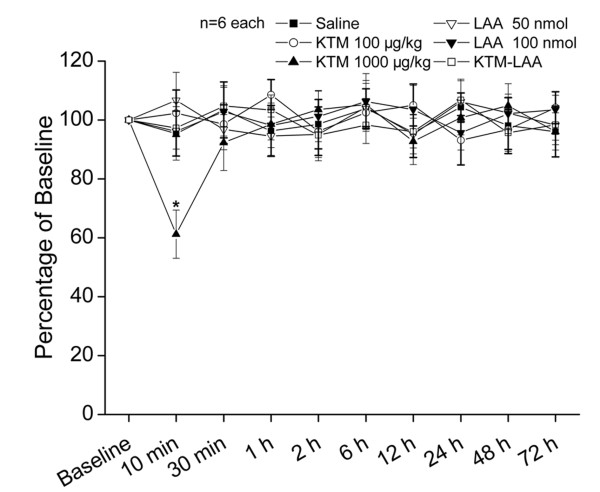
**Effects of ketamine and/or LAA on rats' motor performance in rotarod test**. Immediately after a baseline response was obtained, saline or drugs were administered intrathecally to normal rats and rotarod test was performed at different time points thereafter (6 rats in each group). The score of each group was normalized as a percentage of the baseline value. Compared with baseline, there was no statistical difference obtained from rotarod test after drugs injection, but intrathecally administered 1000 μg/kg of ketamine caused an obvious defect on rotarod test at 10 min after injection. * *P *< 0.05 compared with baseline response.

Nociceptive behavioral results could be easily influenced as a result of motor dysfunctions. In order to assess whether higher dosages of ketamine (100 and 1000 μg/kg) or LAA (50 and 100 nmol) or coadministration (100 μg/kg ketamine and 50 nmol LAA) could produce impairment of motor functions, 36 otherwise experiment-free rats were assessed with the rotarod test. Neither intrathecal ketamine (100 μg/kg) nor LAA (50 nmol or 100 nmol) affected the motor performance of rats compared with the control performance at any time points after injection (Fig 3). Furthermore, coadministration (100 μg/kg ketamine and 50 nmol LAA) didn't influence the motor function either. However, intrathecal ketamine (1000 μg/kg) caused apparent motor dysfunction at 10 min but not other time points after administration.

These results demonstrated that the anti-allodynia effects of ketamine (100 μg/kg), LAA (50 nmol or 100 nmol) or coadministration (100 μg/kg ketamine and 50 nmol LAA) did not impair motor function. Intrathecal ketamine (1000 μg/kg) also exerted effective analgesic effect except the motor impairment at the first 10 min after administration (Fig 3). Therefore, the PWT at 10 min after ketamine (1000 μg/kg) administration was omitted.

### Ketamine or LAA attenuated SNL-induced mechanical allodynia individually in dose-dependent manners

In order to confirm the effects of intrathecal ketamine or LAA on SNL-induced neuropathic pain, we injected these two agents individually with three different concentrations and observed the changes of PWT at different time points after injection on POD 7.

**Figure 4:**
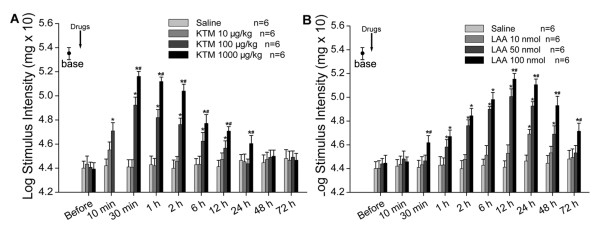
**Effects of intrathecal ketamine or LAA on SNL-induced mechanical allodynia, respectively**. Intrathecal ketamine elevated paw withdrawal thresholds (PWTs) in a dose-dependent manner (A). *, # *P *< 0.05 compared with that of Saline or 100 μg/kg of ketamine, respectively. Intrathecal LAA attenuated SNL-induced mechanical allodynia individually in a dose-dependent manner (B). *, # *P *< 0.05 compared with that of Saline or 50 nmol of LAA, respectively.

Intrathecally administering 10 μg/kg of ketamine could elevate PWT slightly at 10 min after administration, but there were no statistical differences compared to that of SNL-Saline group at any time points (Fig 4A, *P *> 0.05). Intrathecal ketamine (100 μg/kg) elevated PWT significantly 10 min post injection and this anti-allodynia effect lasted to 12 h after administration. Besides, a higher dose of ketamine (1000 μg/kg) elevated PWT apparently 30 min after injection compared to that of saline control and this effect persisted for about 24 h (Fig 4A, *P *< 0.05). We also observed that the anti-allodynia effect of 1000 μg/kg ketamine was better than that of 100 μg/kg ketamine (Fig 4A, *P *< 0.05). All of these results demonstrated that intrathecal ketamine (10, 100 or 1000 μg/kg) showed an effective and reliable anti-allodynia effect in a dose-dependent manner on SNL-induced neuropathic pain, which was a very quick but short effect (Fig 4A).

Intrathecal 10 nmol of LAA could only suppress SNL-induced mechanical allodynia at 24 h after administration (Fig 4B, *P *< 0.05). However, a higher dose of LAA (50 nmol) did elevate PWT at 1 h, with the peak effect occurring at 12 h, and lasted to 48 h after injection, which showed statistical difference with that of SNL-Saline group (Fig 4B, *P *< 0.05). Furthermore, 100 nmol of LAA increased PWT in a more persistent and stronger way compared to that of saline control with its analgesic effects emerged at 30 min, reaching the peak at 12 h, and lasted to 72 h after administration. Intrathecal 100 nmol of LAA produced more significant anti-allodynia effects than that of 50 nmol of LAA (at 30 min, 12 h after administration and thereafter; Fig 4B, *P *< 0.05). These results indicated the dose-related anti-allodynia effect of intrathecal LAA (10, 50 or 100 nmol) on SNL-induced neuropathic pain, which was a late but persistent effect (Fig 4B).

The above results showed that the anti-allodynia effect of ketamine or LAA was significant, dose-related but exhibited individually different effecting patterns: quick but short for ketamine, late but persistent for LAA.

### Effects of combining ketamine with LAA on SNL-induced neuropathic pain

To investigate whether combining NMDAR antagonist ketamine with astrocytic inhibitor LAA could exert some additive and complementary effects on SNL-induced neuropathic pain, we coadministrated ketamine (100 μg/kg) and LAA (50 nmol) simultaneously to SNL rats on POD 7 intrathecally. Then we compared the effect of coadministration (ketamine 100 μg/kg and LAA 50 nmol) with that of individual administration (ketamine 100 μg/kg or LAA 50 nmol).

**Figure 5:**
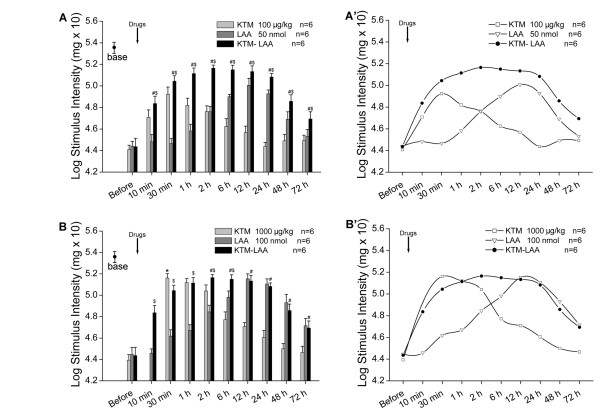
**Effects of combining ketamine with LAA on SNL-induced mechanical allodynia**. PWTs of coadministration group were much higher than that of individual ketamine (100 μg/kg) or LAA (50 nmol) injection at any time points after administration (A). #, $ *P *< 0.05 compared with that of ketamine 100 μg/kg or LAA 50 nmol, respectively. A', Schematic curves according to A implied that compared with that of individual administration, the anti-allodynia effect of coadministration was more quick, stable and persistent. The analgesic effects of coadministration (100 μg/kg of ketamine and 50 nmol of LAA) compared with that of higher doses of individual ketamine (1000 μg/kg) or LAA (100 nmol), respectively (B). * *P *< 0.05 compared with that of Ketamine-LAA group. #, $ *P *< 0.05 compared with that of 1000 μg/kg of ketamine or 100 nmol of LAA, respectively. B', Schematic curves according to B implied that even compared with that of higher doses of individual administration, the anti-allodynia effect of coadministration was still more stable and long-lasting.

The anti-allodynia effect of coadministration appeared at 10 min, reaching the peak at 2 h, and lasted to 72 h after injection. Besides, PWTs of coadministration group were much higher than that of individual ketamine (100 μg/kg) or LAA (50 nmol) injection at any time points after administration (Fig 5A, *P *< 0.05). These results implied that the anti-allodynia effect of coadministration was a very quick, stable and long lasting response (Fig 5A').

Furthermore, we compared the analgesic effects of coadministration (ketamine 100 μg/kg and LAA 50 nmol) with that of intrathecal ketamine (1000 μg/kg) or LAA (100 nmol), respectively. Ketamine (1000 μg/kg) showed stronger analgesic effect only at 30 min after administration than that of coadministration group (Fig 5B, *P *< 0.05). However, there was no statistical difference between coadministration and ketamine 1000 μg/kg at 1 h after administration. Moreover, at 2 h after injection, coadministration showed stronger analgesic effects than that of single ketamine (1000 μg/kg) on PWTs (Fig 5B, *P *< 0.05). Furthermore, coadministration produced stronger effect on PWTs compared with that of LAA (100 nmol) in the first 6 h after administration. However, there were no statistical differences between coadministration and single LAA (100 nmol) at 12 h after administration and thereafter (Fig 5B).

These results suggested that the anti-allodynia effect of coadministration (ketamine 100 μg/kg and LAA 50 nmol) produced a more persistent and effective role than that of individual administration either at mild or high dosage (Fig 5B'). Therefore, we concluded that combining ketamine (100 μg/kg) with LAA (50 nmol) showed some additive and complementary effects on SNL-induced neuropathic pain.

### Effects of ketamine on SNL-induced NMDAR phosphorylation

In order to disclose the underlying mechanisms about the additive and complementary effect of coadministration, Western blot with antibody against pNR1 was performed to detect the NMDAR phosphorylation. The results of behavioral study showed that ketamine exerted strong effect from 30 min to 2 h after given and this effect disappeared at 24 h after administration. Therefore, we chose 2 h and 24 h to check the expression of pNR1.

The results showed that ketamine (100 μg/kg) alone significantly down-regulated pNR1 expression at 2 h after administration compared with that of SNL-Saline group (Fig 2, *P *< 0.05). However, no significant difference could be observed between LAA alone and saline control groups. Drugs coadministration (ketamine 100 μg/kg and LAA 50 nmol) inhibited pNR1 at 2 h after injection, which was obviously lower than that of individual ketamine injection (100 μg/kg) (Fig 2, *P *< 0.05). The NR1 phosphorylation returned to high level at 24 h after ketamine (100 μg/kg) injected alone (*P *> 0.05 compared to SNL-Saline group). However, the level of pNR1 remained low at 24 h after drugs coadministration compared with that of individual ketamine injection or SNL-Saline group (Fig 2, *P *< 0.05).

These results indicated that the anti-allodynia effect of ketamine on neuropathic pain was mainly via suppression of NMDAR function. Moreover, astrocytic inhibitor LAA could enhance the inhibitory effect of ketamine on NMDAR functioning.

### Effects of LAA on SNL-induced astrocytic activation

Immunohistochemistry with antibody against astrocytic specific marker GFAP was performed to detect the astrocytic activation. Behavioral results showed that the effect of intrathecal LAA peaked at 1 d after given and could not persist more than 3 d. So, we chose the time point of 1 d and 3 d to detect GFAP expression.

The results showed that LAA (50 nmol) alone attenuated SNL-induced astrocytic activation at 24 h after administration (Fig 1H), where intrathecal ketamine (100 μg/kg) didn't change SNL-induced microglia activation 1 d after administration (Fig 1G). Furthermore, GFAP expression was obviously down-regulated 24 h after coadministration (ketamine 100 μg/kg and LAA 50 nmol), which was much lower than that of LAA (50 nmol) or ketamine (100 μg/kg) injected alone (Fig 1I). GFAP expression returned to high level at 72 h after LAA (50 nmol) injection (Fig 1L). Meanwhile, intrathecal ketamine (100 μg/kg) didn't show any obvious effects on SNL-induced microglia activation 3 d after application (Fig 1K). However, astrocytic activation was significantly inhibited by drugs coadministration compared to that of intrathecal LAA (50 nmol) or ketamine (100 μg/kg) alone (Fig 1M).

These results suggested that NMDAR antagonist ketamine could facilitate the inhibitory effect of LAA on astrocytic activation.

## Discussion

In the present study, we showed that: 1. SNL induced persistent mechanical allodynia with NMDAR phosphorylation and astrocytic activation in spinal dorsal horn. 2. Intrathecal application of NMDAR antagonist ketamine alleviated mechanical allodynia with decreased NMDAR phosphorylation in a quick but short response, whereas astrocytic cytotoxin LAA relieved mechanical allodynia with attenuated astrocytic activation in a late but persistent manner. 3. Combining ketamine with LAA suppressed neuropathic pain in a quick and stable way, whereas, NMDAR phosphorylation and astrocytic activation were both much more suppressed than those of either single drug administration. Taken together, combination of NMDAR antagonist and astrocytic inhibitor exhibited some additive and complementary analgesic effects on SNL-induced neuropathic pain.

### Effects of ketamine on neuropathic pain

Clinical study shows that intrathecal ketamine is useful for treating neuropathic pain [27] at a sub-anesthetic dosage due to the sedation and other side effects when it is applied at high dose [7]. In the present study, intrathecal ketamine attenuates SNL-induced mechanical allodynia in a dose dependant manner. Spinal NMDAR plays an important role in the development of central sensitization and neuropathic pain via the induction of long-term potentiation (LTP) in dorsal horn nociceptive synaptic transmission. As a functional subunit of NMDAR, NR1 phosphorylation is significantly increased in the ipsilateral spinal cord after nerve injury and coincides with mechanical allodynia [28]. Previous study suggests that ketamine combined with methamphetamine could down-regulate NR1 receptor phosphorylation in rats (phosphorylation site: serine 897) [29]. Moreover, it is reported that NR1 subunit knockout mice are more resistant to ketamine than control wide type mice, indicating that NMDAR NR1 subunit contributes to mediation of ketamine anesthesia or analgesia [30]. The detailed mechanisms underlying ketamine-induced dephosphorylation of NR1 are still unclear. Ketamine could block Ca^2+ ^influx through the NMDA receptor, and then the lowered intracellular Ca^2+ ^decreases the activity of protein kinase C (PKC) and some other intracellular signals [31,32].Inhibition of PKC activity could block capsicin-induced phosphorylation of NR1 [33]. Additionally, PKA activation mediates NR1 phosphorylation in SNL-induced neuropathic pain [28]. Therefore, it is possible that ketamine blocks NMDA receptor and inhibits intracellular PKA, PKC or signals activity, and then decreases NR1 phosphorylation.

In the present study, intrathecal ketamine alleviated mechanical allodynia mainly via suppressing the function of NR1. Therefore, blocking NMDA receptor may be the primary anti-allodynic mechanism of ketamine observed in the present study.

### Effects of the astrocytic specific inhibitor on neuropathic pain

Spinal microglia and astrocytes are both important for the development of neuropathic pain. In SNL-induced neuropathic pain, studies showed that activation of ERK and p38 only presented in spinal microglia at the initiation of neuropathic pain [23,34]. Zhuang *et al *[23] also observed that there was a sequential activation of neurons, microglia and astrocytes following SNL. These results support a role for microglia in the early establishment of SNL-induced neuropathic pain and predict the role for astrocytes in the maintenance of neuropathic pain. Therefore, astrocytes are one of the important cell types for maintenance of SNL-induced neuropathic pain, which is very important for the chronic process of neuropathic pain. Cumulating evidence has shown a critical role for activated astrocytes in nerve injury-induced neuropathic pain [35]. Ultrastructural evidence suggests that cell degeneration and death are confined to astrocytes after the injection of LAA into the striatum [36]. Consistent with a previous study [21], we demonstrated that LAA relieved late phase mechanical allodynia in a dose dependent manner. In the present experiment, we injected LAA and observed that SNL-induced mechanical allodynia was suppressed and GFAP expression was down regulated. These results indicated that inhibition of astrocytic activation may be a useful way to conquer neuropathic pain, which may be a complementary option to modern neuronal based therapeutics.

### Drugs combination

The clinical concept of balanced or associative manner proposes to use a combination of analgesics and other treatments to provide better pain relief and minimized side effects [8-10]. However, all of them only concerned about the neuronal participation in the neuropathic pain states. Spinal astrocytes play important roles in the development of SNL-induced neuropathic pain. Therefore, combining ketamine with the astrocytic inhibitor may provide a potential strategy for treating neuropathic pain.

In the present study, we observed that combination of ketamine and LAA showed additive and complementary effects of anti-allodynia. The analgesic effect of coadministration appeared earlier than that of intrathecal ketamine alone. Moreover, the peak effect of coadministration was much stronger than that of individual ketamine or LAA injection. Furthermore, the combination of drug administration exerted more powerful inhibition on NR1 phosphorylation and astrocytic activation. Our results suggest that combination of NMDAR antagonist ketamine and astrocytic cytotoxin LAA is an effective way to relieve SNL-induced mechanical allodynia. Then we further search for the underlying mechanisms of this effect.

Intrathecal ketamine enhanced the analgesic effect of LAA on tactile allodynia and potentiated the inhibitory effect of LAA on astrocytic activation. As observed in our previous study, inhibiting astrocytic activation is probably a novel analgesic mechanism of ketamine [37]. Some opioid receptors (μ, κ and δ subtypes) and toll like receptors (TLRs) are expressed on astrocytes. It is shown that ketamine can function on opioid receptors [38,39] and TLRs [40-42] and thus inhibiting astrocytic activation. Besides, nitric oxide (NO) released following NMDAR activation could induce astrocytic activation [43]. Consequently, blocking NMDAR-NO pathway probably prevents astrocytic activation. All of these evidence supported our results that intrathecal ketamine facilitated the effects of astrocytic inhibitor LAA both on anti-allodynia and GFAP down-regulation.

On the other hand, our study observed that astrocytic toxin LAA could facilitate the effects of ketamine on neuropathic pain relief and reduction of pNR1 level. Studies indicated that some "gliotransmitters", proinflammatory cytokine and chemokine released from activated astrocytes could facilitate neuronal activity [20,44-46]. Therefore, blocking astrocytic activation might dephosphorylate NR1. Recent studies show that glutamate exocytose from astrocytes enhanced synaptic strength at excitatory synapses [45,47]. This effect is mainly mediated by neuronal NMDAR. Therefore, inhibiting astrocytic activation (e.g. with LAA in our study) could down-regulate activation of NMDAR via preventing glutamate release from astrocytes. In addition, emerging literature implicates a role for glia-cytokines-neurons in persistent pain [12]. Nerve injury induces astrocytic activation and release of some proinflammatory cytokines. It is confirmed that pain-induced up-regulation of IL-1β is selectively localized to astrocytes, while IL-1 receptor (IL-1R) is exclusively expressed on neurons [12,48]. Furthermore, it is showed that IL-1R and NR1 are co-localized in spinal neurons [48]. Applying IL-1R antagonist and glial inhibitor, attenuates NMDAR phosphorylation [48]. In vitro application of IL-1β induces NR1 phosphorylation, which is blocked by an IL-1R antagonist [12]. These results further provide evidence for our study that LAA could decrease pNR1, and thus enhances the role of ketamine.

LAA is a kind of toxin and cannot be used as a clinical drug although it can produce significant analgesic effects. However, in the present study, LAA is used to confirm the active participation of astrocytes in neuropathic pain. More importantly, we want to provide a potential strategy on the treatment of neuropathic pain. It is expected that in the future, some analgesic treatments targeting astrocytes with limited side effects will be developed.

In conclusion, the results of the present study show that ketamine and LAA individually present anti-allodynic effects in neuropathic pain, whereas their coadministration exhibits additive and complementary effects on mechanical allodynia. This combination of these two drugs provided a quick, stable and enhanced effect, with less side effects of ketamine in current treatment. Although LAA is now just used in experimental study but not clinical research or therapy, our results provide a new potential strategy for treating clinical neuropathic pain.

## Methods

### Animals preparation

Male *Sprague-Dawley *rats (180-200 g) were housed in plastic cages, and maintained on a 12:12 h light/dark cycle under conditions of 22-25°C ambient temperature with food and water available. Totally 160 rats contributed to the whole experiments. All experimental procedures received prior approval from the Animal Use and Care Committee for Research and Education of the Fourth Military Medical University (Xi'an, China), and the ethical guidelines to investigate experimental pain in conscious animals [49]. All efforts were made to minimize animal suffering and to reduce the number of animals used.

### Intrathecal implantation

Intrathecal implantation was performed by inserting polyethylene (PE) tubing to inject the drug directly into the subarachnoid space of the lumbar enlargement. Briefly, a midline incision (3 cm) was made at the back of the rat at the level of the thoracic vertebrae, under pentobarbital anesthesia (45 mg/kg, *i.p*.). A pre-measured length of PE-10 tubing (I.D. 0.28 mm and O.D. 0.61 mm) was passed caudally from the T8 to the L3 level of the spinal cord, and 2 cm of the free ending was left exposed in the upper thoracic region. Rats were allowed to recover for a 3-5 d before further use. Only the animals judged as neurologically normal and that showed complete paralysis of the tail and bilateral hind legs after administration of 2% lidocaine (10 μl) through the intrathecal catheter were used for the following experiments.

### SNL

To create the rat SNL model, under pentobarbital anesthesia (45 mg/kg, *i.p*.), the left transverse process of the L5 vertebra was first removed to expose the L4 and L5 spinal nerves. The L5 spinal nerve was then carefully isolated and tightly ligated with 6-0 silk thread [50]. The surgical procedure for the sham group was identical to that of the SNL group, except that the spinal nerve was not ligated. Animals were raised for 1 w till the intrathecal drugs administration on post operative day (POD) 7.

### Intrathecal drugs administration

The astrocytic cytotoxin LAA (Sigma, St. Louis, MO, USA) and S(+)-ketamine hydrochloride (Sigma) were dissolved and diluted with preservative-free normal saline solution for administration. Normal saline (0.9%) was used as the negative control. Animals were divided into 5 groups for administration: Sham-Saline group (n = 6, a volume of 10 μl normal saline was injected into sham rats), SNL-Saline group (n = 6, a volume of 10 μl normal saline was injected into SNL rats), SNL-Ketamine group (n = 6 for each 3 subgroups; 10 μl of 10, 100 or 1000 μg/kg ketamine was injected into SNL rats, respectively), SNL-LAA group (n = 6 for each 3 subgroups; 10 μl of 10, 50 or 100 nmol LAA was injected into SNL rats, respectively), SNL-Ketamine-LAA group (n = 6; 50 nmol LAA together with 100 μg/kg ketamine in a total volume of 10 μl was injected into SNL rats, respectively). Drugs and saline were injected intrathecally over 30 s, followed by a 10 μl flush of normal saline.

### Nociceptive behavioral tests

Animals were habituated to the testing environment for 3 d before baseline testing, and then were placed under inverted plastic boxes (30 × 30 × 50 cm^3^) on an elevated mesh floor and allowed to habituate for 30 min before the threshold testing. Briefly, a logarithmic series of 8 calibrated Semmes-Weinstein monofilaments (von-Frey hairs; Stoelting, Kiel, WI, USA) were applied to the ipsilateral hindpaws to determine the stimulus intensity threshold stiffness required to elicit a paw withdrawal response. Log stiffness of the hairs is determined by log10 (milligrams × 10) [17]. The 8 filaments had the following log-stiffness values (value in grams is given in parentheses): 4.17 (1479 mg), 4.31 (2041 mg), 4.56 (3630 mg), 4.74 (5495 mg), 4.93 (8511 mg), 5.07 (11749 mg), 5.18 (15136 mg), and 5.46 (28840 mg). The range of monofilaments (1.479-28.840 gm) produced a logarithmically graded slope when interpolating a 50% response threshold of stimulus intensity (expressed as log10 (milligrams × 10)) [51]. Assessments were made before surgery for baseline value. Then on POD 7, behavioral tests were performed 30 min before, and 10 min, 30 min, 60 min, 2 h, 6 h, 12 h, 24 h, 48 h, and 72 h after drug administration, respectively. The behavioral responses were used to calculate the 50% PWT, by fitting a Gaussian integral psychometric function using a maximum-likelihood fitting method, as described in detail previously [17]. This fitting method allowed parametric statistical analysis. All the PWT tests were performed in a double-blind manner.

### Rotarod test

In order to assess whether the drugs used in the present experiment could influence motor function, which might influence the behavioral results, we performed rotarod tests on drug administered but operation- and behavioral observation-free rats. Rats with no previous exposures to the rotarod test were placed on the Ugo Basile 7650 Rotarod accelerator treadmill (Ugo Basile, Varese, Italy) set at the minimal speed for training sessions of 1-2 min at intervals of 30-60 min. After this learning period, the animals were placed on to the rotarod at a constant speed of 25 *RPM*. As the animal took a grip of the drum, the accelerator mode was selected on the treadmill, *i.e*. the rotation rate of the drum was increased linearly at 20 *RPM*. Thereafter, the time was measured from the start of the acceleration period until the rat fell off the drum. The cut-off time was 30 s. Each rat was tested 30 min before as control performance and at 10 min, 30 min, 60 min, 2 h, 6 h, 12 h, 24 h, 48 h, and 72 h after drugs administration, respectively. The time that the animal remained on the rotarod was recorded and expressed as a percentage of that animal's own mean control performance.

### Immunohistochemistry

After deeply anesthesia with pentobarbital (60 mg/kg, *i.p*.), the rats were perfused through the ascending aorta with 100 ml 0.9% saline followed by 500 ml 0.1 M phosphate buffer (PB, pH 7.3) that contained 4% paraformaldehyde and 2% picric acid. After perfusion, the L5 spinal segment was removed and post-fixed in the same fixative for 2-4 h and then cryoprotected for 24 h at 4°C in 0.1 M PB that contained 30% sucrose. Transverse frozen spinal sections (30 μm thick) were cut with a cryostat (Leica CM1800; Heidelberg, Germany) and collected serially in three dishes. Each dish contained a complete set of serial sections that were processed for immunofluorescent staining. One of the dishes was selected randomly. The sections in the dish were rinsed in 0.01 M phosphate-buffered saline (PBS, pH 7.3) three times (10 min each), blocked with 2% goat serum in 0.01 M PBS that contained 0.3% Triton X-100 for 1 h at room temperature (RT, 20-25°C), and then used for immunofluorescent staining. The sections were incubated overnight at 4°C with primary antibody: mouse anti-GFAP (1:5000; Chemicon, Temecula, CA, USA). The sections were washed three times in 0.01 M PBS (10 min each) and then incubated for 4 h at RT with the secondary antibody: FITC-conjugated horse anti-mouse IgG (1:500; Vector, Burlingame, CA, USA). The specificity of the staining was tested on the sections in another dish by omission of the primary specific antibodies. No immunoreactive products were found on the sections (data not shown). Confocal images were obtained using a confocal laser microscope (FV1000; Olympus, Tokyo, Japan) and digital images were captured with Fluoview 1000 (Olympus). For semi-quantification, the fluorescent brightness value of GFAP-like immunoreactivity was detected on the same areas of the dorsal horn by using software under IX-70 confocal microscope. After the images were captured, optical density (OD) of the same areas of the ipsilateral superficial dorsal horn (laminae I and II, Fig 1K) was calculated and averaged across the five spinal sections [25]. The relative value of GFAP immunodensity was expressed as percentage changes compared to the Sham-Saline control.

### Western blot

Animals were rapidly sacrificed and the L5 dorsal horns were rapidly removed and frozen on dry ice for western blot. The spinal dorsal horn was dissected using the "open book" method [23]. Briefly, the L5 spinal cord segment was dissected according to the termination of the L4 and L5 dorsal roots. Then, the spinal segment was cut into a left and right half from the midline. Finally, the left half was further split into the dorsal and ventral horns at the level of the central canal. The selected region was homogenized with a hand-held pestle in SDS sample buffer (10 ml mg^-1 ^tissue), which contained a cocktail of proteinase and phosphatase inhibitors. The electrophoresis samples were heated at 100°C for 5 min and loaded onto 10% SDS-polyacrylamide gels with standard Laemmli solutions (Bio-Rad Laboratories, CA, USA). The proteins were electroblotted onto a polyvinylidene difluoride membrane (PVDF, Immobilon-P, Millipore, Billerica, MA, USA). The membranes were placed in a blocking solution, which contained Tris-buffered saline with 0.02% Tween (TBS-T) and 5% non-fat dry milk, for 1 h, and incubated overnight under gentle agitation with primary antibodies: mouse anti-pNR1 (1: 500; Sigma) and rabbit anti-β-actin (1: 1000; Sigma). Bound primary antibodies were detected with the anti-rabbit or anti-mouse horseradish peroxidase (HRP)-conjugated secondary antibody (1:10000; Amersham Pharmacia Biotech Inc., Piscataway, NJ, USA). Between each step, the immunoblots were rinsed with TBS-T. All reactions were detected by the enhanced chemiluminescence (ECL) detection method (Amersham). The densities of protein blots were analyzed by using Labworks Software (Ultra-Violet Products, UK). The densities of pNR1 and β-actin immunoreactive bands were quantified with background subtraction. The same size of square was drawn around each band to measure the density and the background near that band was subtracted. Since β-actin levels did not change significantly after inflammation and nerve injury [12], we used β-actin levels as a loading control, and pNR1 levels were normalized against β-actin levels and expressed as percentage changes compared to the Sham-Saline control.

### Quantification and statistical analysis

All data were collected by researchers blinded to the surgery and reagents used. Data from western blot and immunohistochemistry were expressed as mean ± SD. Differences in changes of values over time of each group were tested using one-way ANOVA, followed by the least significant difference test. Data from the von-Frey test were presented as mean ± SD and analyzed as the interpolated 50% threshold (absolute threshold) in log base 10 of stimulus intensity (monofilament stiffness in milligrams × 10). Repeated measures ANOVA (with Bonferroni confidence interval adjustment) was used and conducted for analyzing. Data from the rotarod test were presented as mean ± SD. Repeated measures ANOVA (with Bonferroni confidence interval adjustment) was used and conducted for analyzing. All statistical analyses were performed using SPSS^® ^version 16.0 software (SPSS Inc., Chicago, IL, USA). *P *< 0.05 was considered statistically significant.

## Competing interests

The authors declare that they have no competing interests.

## Authors' contributions

XPM and WW (Wei Wang) performed the animal surgery, carried out the Western blot study and drafted the manuscript. TZ carried out the immunofluorescence. CZ and LC performed the behavioral test. WW (Wen Wang) participated in producing graphics and performed the statistical analysis. LXX, SXW and YQL conceived of the study, and participated in its design and coordination. All authors read and approved the final manuscript.
